# ProtNN: fast and accurate protein 3D-structure classification in structural and topological space

**DOI:** 10.1186/s13040-016-0108-2

**Published:** 2016-09-23

**Authors:** Wajdi Dhifli, Abdoulaye Baniré Diallo

**Affiliations:** Department of Computer Science, University of Quebec At Montreal, PO box 8888, Downtown stationMontreal, H3C 3P8 Canada

**Keywords:** Protein 3D-structure, Protein classification, Graph classification

## Abstract

**Background:**

Studying the functions and structures of proteins is important for understanding the molecular mechanisms of life. The number of publicly available protein structures has increasingly become extremely large. Still, the classification of a protein structure remains a difficult, costly, and time consuming task. The difficulties are often due to the essential role of spatial and topological structures in the classification of protein structures.

**Results:**

We propose ProtNN, a novel classification approach for protein 3D-structures. Given an unannotated query protein structure and a set of annotated proteins, ProtNN assigns to the query protein the class with the highest number of votes across the *k* nearest neighbor reference proteins, where *k* is a user-defined parameter. The search of the nearest neighbor annotated structures is based on a protein-graph representation model and pairwise similarities between vector embedding of the query and the reference protein structures in structural and topological spaces.

**Conclusions:**

We demonstrate through an extensive experimental evaluation that ProtNN is able to accurately classify several datasets in an extremely fast runtime compared to state-of-the-art approaches. We further show that ProtNN is able to scale up to a whole PDB dataset in a single-process mode with no parallelization, with a gain of thousands order of magnitude in runtime compared to state-of-the-art approaches.

## Introduction

Proteins are ubiquitous in the living cells. They play key roles in the functional and evolutionary machinery of species. Studying protein functions and structures is paramount for understanding the molecular mechanisms of life. High-throughput technologies are yielding millions of protein-encoding sequences that currently lack any functional characterization [1–3]. The number of proteins in the Protein Data Bank (PDB) [4] has more than tripled over the last decade. Alternative databases such as SCOP [5] and CATH [6] are undergoing the same trend. However, the classification of protein structures remains a difficult, costly, and time consuming task. Manual protein classification methods are no longer able to follow the rapid increase of data. Accurate computational and machine learning tools present an efficient alternative that could offer considerable boosting to meet the increasing load of data.

Proteins are composed of complex three-dimensional folding of long chains of amino acids. This spatial structure is an essential component in protein functionality and is thus subject to evolutionary pressures to optimize the inter-residue contacts that support it [7]. Existing computational methods for protein classification try to simulate biological phenomena that define the structure and function of a protein. The most conventional technique is to perform a similarity search between an unknown protein and a reference database of annotated proteins. The query protein is assigned with the same class of the most similar (based on the sequence or the structure) reference protein. There exists several classification methods based on the protein sequence (*e*.*g*. Blast [8], ProtFun [9], SVM-Prot [10, 11] …); or on the protein structure (*e*.*g*. Combinatorial Extension [12], Sheba [13], FatCat [14], Fragbag [15], …). These methods rely on the assumption that proteins sharing the most common sites are more likely to belong to the same class. This classification strategy is based on the hypothesis that structurally similar proteins could share a common ancestor [16]. Another popular approach for protein functional classification is to look for relevant subsequences or substructures (also so-called motifs) among known proteins, then use them as features to classify unknown proteins. Such motifs could be discriminative [17], representative [18], cohesive [7], *etc*. Each of the mentioned protein classification approaches suffers different drawbacks. Sequence (and subsequences)-based classification do not incorporate spatial information of amino acids that are not contiguous in the primary structure but interconnected in 3D space. This makes them less efficient in the classification of structurally similar proteins with low sequence similarity (remote homologues). Both structure and substructure-based classification techniques do incorporate spatial information which makes them more efficient than sequence-based classification. However, such consideration makes these methods subject to the *“no free lunch”* principle [19], where the gain in accuracy comes with an offset of computational cost. Hence, it is essential to find an efficient way to incorporate 3D-structure information with low computational complexity.

In this paper, we present PROTNN, a novel approach for protein 3D-structure classification. PROTNN incorporates protein 3D-structure information via the combination of a rich set of structural and topological descriptors. This guarantees an informative multi-view representation of the structure that considers spatial information through different dimensions. Such a representation transforms the complex protein 3D-structure into an attribute-vector of fixed size which guarantees the computational efficiency. For classification, PROTNN assigns to a query protein the class having the highest number of votes across the set of its *k* most similar reference proteins, where *k* is a user-defined parameter. Experimental evaluation shows that PROTNN is able to accurately classify different benchmark datasets with a gain of up to 47x of computational cost compared to gold standard approaches from the literature such as Combinatorial Extension [12] and FatCat [14]. We further show that PROTNN is able to scale up to a PDB-wide dataset in a single-process mode with no parallelization, where it outperformed state-of-the-art approaches with thousands order of magnitude in runtime on classifying a 3D-structure against the entire PDB.

## Methods

### Graph representation of protein 3D-structures

A crucial step in computational studies of protein 3D-structures is to look for a convenient representation of their spatial conformations. Graphs represent the most appropriate data structures to model the complex structures of proteins. In this context, a protein 3D-structure can be seen as a set of elements (amino acids and atoms) that are interconnected through chemical interactions [7, 16, 18, 20]. These interactions are mainly:

- Covalent bonds between atoms sharing pairs of valence electrons,
- Ionic bonds of electrostatic attractions between oppositely charged components,
- Hydrogen bonds between two partially negatively charged atoms sharing a partially positively charged hydrogen,
- Hydrophobic interactions where hydrophobic amino acids in the protein closely associate their side chains together,
- Van der Waals forces which represent transient and weak electrical attraction of one atom for another when electrons are fluctuating.

**Fig. 1:**
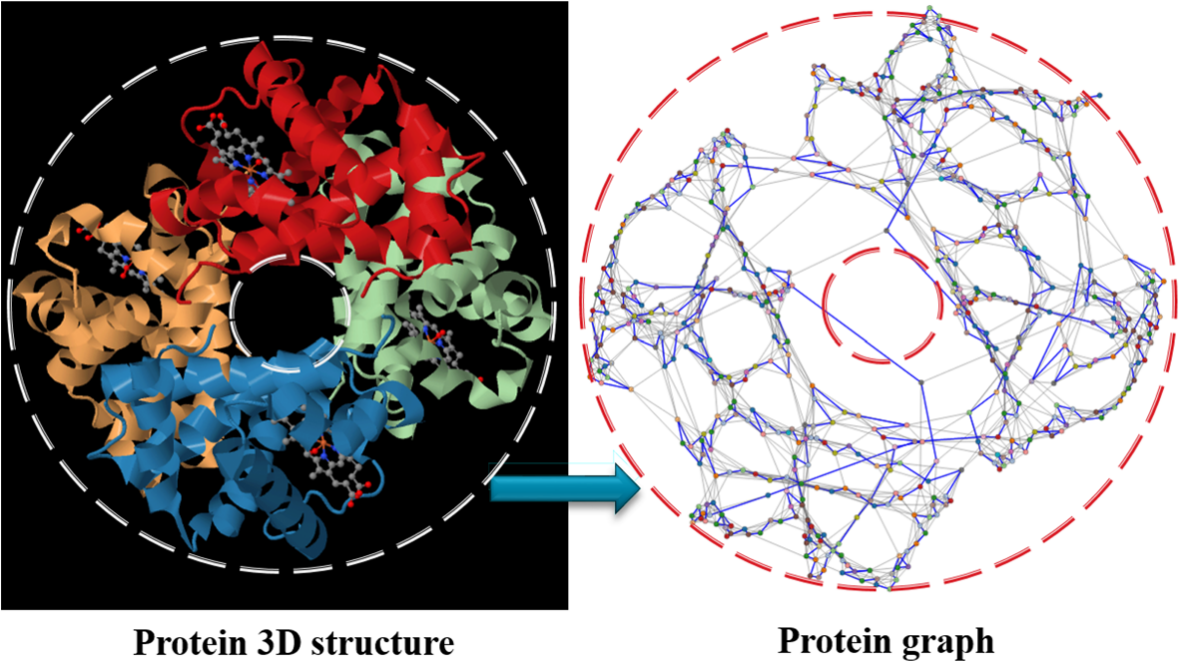
The human hemoglobin protein 3D-structure (PDBID: 1GZX) and its corresponding graph representation. Nodes and edges represent, respectively, amino acids from the structure and links between them. *Blue edges* represent links from the primary structure and *gray edges* are spatial links between distant amino acids

These chemical interactions are supposed to be the analogues of graph edges. Figure 1 shows a real example of the human hemoglobin protein and its graph representation. The Figure shows clearly that the graph representation preserves the overall structure of the protein and its components.

**Protein Graph Model:** Let *G* be a graph consisting of a set of nodes *V* and edges *E*. *L* is a label function that associates a label *l* to each node in *V*. Each node of *G* represents an amino acid from the 3D-structure, and is labeled with its corresponding amino acid type. Let *Δ* be a function that computes the euclidean distance between pairs of nodes *Δ*(*u*,*v*),∀*u*,*v*∈*V*, and *δ* a distance threshold. Each node in *V* is defined by its 3D coordinates in IR^3^, and both *Δ* and *δ* are expressed in angstroms (Å). Two nodes *u* and *v* (∀*u*,*v*∈*V*) are linked by an edge *e*(*u*,*v*)∈*E*, if the distance between their *C*_*α*_ atoms is below or equal to *δ*. Formally, the adjacency matrix *A* of *G* is defined as follows:

1 $$ A_{u,v}= \left\{\begin{array}{ll} 1,~if~\Delta(C_{\alpha_{u}}, C_{\alpha_{v}})\leq \delta \\ 0,~otherwise \end{array}\right.  $$

### Structural and topological embedding of protein graphs

#### Graph embedding

Graph-based representations are broadly used in multiple application fields including bioinformatics [16, 18, 21]. However, they suffer major drawbacks with regards to processing tools and runtime. Graph embedding into vector spaces is a very popular technique to overcome both drawbacks [21]. It aims at providing a feature vector representation for every graph, allowing to bridge the gap between the representational power of graphs, the rich set of algorithms that are available for feature-vector representations, and the need for rapid processing algorithms to handle the massively available biological data. In PROTNN, each protein 3D-structure is represented by a graph according to Eq. 1. Then, each graph is embedded into a vector of structural and topological features under the assumption that structurally similar graphs should give similar structural and topological feature-vectors. In such manner, PROTNN guarantees accuracy and computational efficiency.

#### Structural and topological attributes

In order to avoid the loss of structural information in the embedding and to guarantee PROTNN accuracy, we use a rich set of structural and topological attributes from the literature that have shown to be interesting and efficient in describing connected graphs [22–27]. It is important to mention that this list could be extended as needed. In the following, we list the set of attributes that are used in PROTNN:

1. **Number of nodes**: The total number of nodes of the graph, |*V*|.
2. **Number of edges**: The total number of edges of the graph, |*E*|.
3. **Average degree**: The degree of a node *u*, denoted *deg*(*u*), is the number of its adjacent nodes. The average degree of a graph *G* is the average of all *deg*(*u*), ∀*u*∈*G*. Formally: $ deg(G) = \frac {1}{\mid V\mid } \sum ^{\mid V\mid }_{i=1} deg(u_{i})$.
4. **Density**: The density of a graph *G*=(*V*,*E*) measures how many edges are in *E* compared to the number of maximum possible edges between the nodes in *V*. Formally: $ den(G) = \frac {2 \mid E\mid }{(\mid V\mid \ast (\mid V\mid -1))}$.
5. **Average clustering coefficient**: The clustering coefficient of a node *u*, denoted *c*(*u*), measures how complete the neighborhood of *u* is, $c(u)= \frac {2 e_{u}}{k_{u} (k_{u} - 1)}$ where *k*_*u*_ is the number of neighbors of *u* and *e*_*u*_ is the number of connected pairs of neighbors. The average clustering coefficient of a graph *G*, is given as the average value over all of its nodes. Formally: $C(G)= \frac {1}{\mid V\mid } \sum _{i=1}^{\mid V\mid } c(u_{i})$.
6. **Average effective eccentricity**: For a node *u*, the effective eccentricity represents the maximum length of the shortest paths between *u* and every other node *v* in *G*, *e*(*u*)=*max*{*d*(*u*,*v*):*v*∈*V*,*u*≠*v*}, where *d*(*u*,*v*) is the length of the shortest path from *u* to *v*. The average effective eccentricity is defined as $Ae(G)= \frac {1}{\mid V\mid }\sum _{i=1}^{\mid V\mid } e(u_{i})$.
7. **Effective diameter**: It represents the maximum value of effective eccentricity over all nodes in the graph *G*, *i*.*e*., *diam*(*G*)=*max*{*e*(*u*)∣*u*∈*V*} where *e*(*u*) represents the effective eccentricity of *u* as defined above.
8. **Effective radius**: It represents the minimum value of effective eccentricity over all nodes of *G*, *rad*(*G*)=*min*{*e*(*u*)∣*u*∈*V*}.
9. **Closeness centrality**: The closeness centrality measures how fast information spreads from a given node to other reachable nodes in the graph. For a node *u*, it represents the reciprocal of the average shortest path length between *u* and every other reachable node in the graph *G*, $C_{c}(u) = \frac {{\mid V\mid }-1}{\sum _{v\in \lbrace V\setminus u\rbrace } d(u,v)}$ where *d*(*u*,*v*) is the length of the shortest path between the nodes *u* and *v*. For *G*, we consider the average value of closeness centrality of all its nodes, $C_{c}(G) = \frac {1}{\mid V\mid } \sum _{i=1}^{\mid V\mid } C_{c}(u_{i})$.
10. **Percentage of central nodes**: It is the ratio of the number of central nodes from the number of nodes in the graph. A node *u* is central if the value of its eccentricity is equal to the effective radius of the graph, *e*(*u*)=*rad*(*G*).
11. **Percentage of end points**: It represents the ratio of the number of nodes with *deg*(*u*)=1 from the total number of nodes of *G*.
12. **Number of distinct eigenvalues**: A scalar $\leftthreetimes $ is called an *eigenvalues* of a squared matrix *M* if there exists an *eigenvectorx* such that $Mx = \leftthreetimes x$. The adjacency matrix *A* of *G* has a set of eigenvalues. We count the number of distinct eigenvalues of *A*.
13. **Spectral radius**: Let $\leftthreetimes _{1}, \leftthreetimes _{2},..., \leftthreetimes _{m}$ be the set of eigenvalues of the adjacency matrix *A* of *G*. The spectral radius of *G*, denoted *ρ*(*G*), represents the largest magnitude eigenvalue, *i*.*e*., $\rho (G) = max(\mid \leftthreetimes _{i}\mid)$ where *i*∈{1,..,*m*}.
14. **Second largest eigenvalue**: The value of the second largest eigenvalue.
15. **Energy**: The energy of an adjacency matrix *A* of a graph *G* is defined as the squared sum of the eigenvalues of *A*. Formally: $E(G) = \sum ^{m}_{i=1}{\leftthreetimes _{i}^{2}}$.
16. **Neighborhood impurity**: For a node *u* having a label *L*(*u*) and a neighborhood *N*(*u*), it is defined as *ImpNeigh*(*u*)=∣*L*(*v*):*v*∈*N*(*u*),*L*(*u*)≠*L*(*v*)∣. The neighborhood impurity of *G* is the average *ImpNeigh* over all nodes.
17. **Link impurity**: An edge {*u*,*v*} is considered to be impure if *L*(*u*)≠*L*(*v*). The link impurity of a graph *G* with |*E*| edges is defined as: $\frac {\mid \{u,v\}\in E: L(u)\neq L(v)\mid }{\mid E\mid }$.
18. **Label entropy**: It measures the uncertainty of labels. For a graph *G* of *k* labels, it is defined as $E(G)= -\sum _{i=1}^{k} p(l_{i})\textit {log }p(l_{i})$, where *l*_*i*_ is the *i*^*th*^ label.

#### Complexity

The computational complexity of the structural and topological attributes differ from one attribute to another. Some of the attributes are very easy to compute like the number of nodes and the number of edges which are respectively computed in $\mathcal {O}(n)$ and $\mathcal {O}(e)$ where *n* is the number of nodes and *e* is that of edges in the graph. The density of the graph can directly computed from the number of nodes and that of edges. The average degree can be computed in $\mathcal {O}(n+e)$. Some other attributes are more complex to compute and thus require higher computational runtime. The average clustering coefficient can be computed in the $\mathcal {O}(n^{2})$. The average effective eccentricity, the effective diameter, the effective radius, the closeness centrality, and the percentage of end points are all computed based on the set of shortest paths between all pairs of nodes of the graph. For each node, the shortest path can be computed in $\mathcal {O}(n+e)$ and thus in $\mathcal {O}(n^{2}+ne)$ for all nodes of the graph. The percentage of end points can directly be computed in $\mathcal {O}(n+e)$. The number of distinct eigenvalues, the spectral radius, the second largest eigenvalue, and the energy are all computed based on the eigenvalue decomposition of the graph which is upper bounded by $\mathcal {O}(n^{3})$ in the worst case. However, for sparse graphs it can be computed in less time. The computation of the neighborhood impurity is upper bounded by $\mathcal {O}(nk)$, where *k* is the largest node degree in the graph. The link impurity and label entropy can respectively be computed in $\mathcal {O}(n+e)$ and $\mathcal {O}(n)$.

### PROTNN: nearest neighbor protein functional classification

We propose PROTNN, a protein structure classification approach based on the principal of the k-nearest neighbor algorithm [28]. The general classification pipeline of PROTNN can be described as follows: first a preprocessing is performed on the reference protein database *Ω* in which a graph model *G*_*P*_ is created for each reference protein *P*, ∀*P*∈*Ω*, according to Eq. 1. A structural and topological description vector *V*_*P*_ is created for each graph model *G*_*P*_, by computing the corresponding values of each of the structural and topological attributes described in Section “Structural and topological attributes”. The resulting matrix $M_{\Omega } = \bigcup V_{P}$, ∀*P*∈*Ω*, represents the preprocessed reference database that is used for prediction in PROTNN. In order to guarantee an equal participation of all used attributes in the classification, a min-max normalization ($x_{normalized} = \frac {x - min}{max-min}$, where *x* is an attribute value, *min* and *max* are the minimum and maximum values for the attribute vector) is applied on each attribute of *M*_*Ω*_ independently such that no attribute will dominate in the prediction. It is also worth mentioning that for real world applications *M*_*Ω*_ is computed once, and it can be incrementally updated with other attributes as well as newly added protein 3D-structures with no need to recompute the attributes for the entire set. This guarantees a high flexibility and easy extension of PROTNN in real world application.



The prediction step in PROTNN is described in Algorithm 1. In prediction, a query protein 3D-structure *Q* with an unknown function, is first transformed into its corresponding graph model *G*_*Q*_. The structural and topological attributes are computed for *G*_*Q*_ forming its query description vector *V*_*Q*_. The query protein *Q* is scanned against the entire reference database *Ω*, where the distance between *V*_*Q*_ and each of the reference vectors ∀*V*_*P*_∈*M*_*Ω*_ is computed and stored in *Vdist*_*Q*_, with respect to a distance measure. The *k* most similar reference proteins *NN*$_{Q}^{k}$ are selected, and the query protein *Q* is predicted to belong to the class with the highest number of votes across the set of *NN*$_{Q}^{k}$ reference proteins, where *k* is user-defined.

### Datasets

#### Benchmark datasets

To assess the classification performance of PROTNN, we performed an experiment on six well-known benchmark datasets of protein structures that have previously been used in [17, 29–31]. Each dataset is composed of positive protein examples that are from a selected protein family, and negative protein examples that are randomly sampled from the PDB [4]. Table 1 summarizes the characteristics of the six datasets. SCOP ID, Family name, Pos., Neg., Avg. ∣*V*∣, Avg. ∣*E*∣, Max. ∣*V*∣ and Max. ∣*E*∣ correspond respectively to the identifier of the positive protein family in SCOP, its name, the number of positive examples, the number of negative examples, the average number of nodes, the average number of edges, the maximal number of nodes and the maximal number of edges in each dataset. The selected positive protein families are Vertebrate phospholipase A2, G-protein family, C1-set domains, C-type lectin domains, Proteasome subunits and Protein kinases, catalytic subunits.

**Table 1:** Characteristics of the experimental datasets

Dataset	SCOP ID	Family name	Pos.	Neg.	Avg. ∣*V*∣	Avg. ∣*E*∣	Max. ∣*V*∣	Max. ∣*E*∣
DS1	48623	Vertebrate phospholipase A2	29	29	160	628	451	1812
DS2	52592	G-proteins	33	33	246	971	897	3544
DS3	48942	C1-set domains	38	38	238	928	768	2962
DS4	56437	C-type lectin domains	38	38	185	719	775	3016
DS5	56251	Proteasome subunits	35	35	231	929	897	3544
DS6	88854	Protein kinases, catalyc subunits	41	41	275	1077	775	3016

SCOP ID, Family name, Pos., Neg., Avg. ∣*V*∣, Avg. ∣*E*∣, Max. ∣*V*∣ and Max. ∣*E*∣ correspond respectively to the identifier of the positive protein family in SCOP, its name, the number of positive examples, the number of negative examples, the average number of nodes, the average number of edges, the maximal number of nodes and the maximal number of edges in each dataset

##### Vertebrate phospholipase A2:

Phospholipase A2 are enzymes from the class of hydrolase, which release the fatty acid from the hydroxyl of the carbon 2 of glycerol to give a phosphoglyceride lysophospholipid. They are located in most mammalian tissues.

##### G-proteins:

G-proteins are also known as guanine nucleotide-binding proteins. These proteins are mainly involved in transmitting chemical signals originating from outside a cell into the inside of it. G-proteins are able to activate a cascade of further signaling events resulting a change in cell functions. They regulate metabolic enzymes, ion channels, transporter, and other parts of the cell machinery, controlling transcription, motility, contractility, and secretion, which in turn regulate diverse systemic functions such as embryonic development, learning and memory, and homeostasis.

##### C1-set domains:

The C1-set domains are immunoglobulin-like domains, similar in structure and sequence. They resemble the antibody constant domains. They are mostly found in molecules involved in the immune system, in the major histocompatibility complex class I and II complex molecules, and in various T-cell receptors.

##### C-type lectin domains:

Lectins occur in plants, animals, bacteria and viruses. The C-type (Calcium-dependent) lectins are a family of lectins which share structural homology in their high-affinity carbohydrate-recognition domains. This dataset involves groups of proteins playing diverse functions including cell-cell adhesion, immune response to pathogens and apoptosis.

##### Proteasome subunits:

Proteasomes are critical protein complexes that primarily function to breakdown unneeded or damaged proteins. They are located in the nucleus and cytoplasm. The proteasome recycles damaged and misfolded proteins as well as degrades short-lived regulatory proteins. As such, it is a critical regulator of many cellular processes, including the cell cycle, DNA repair, signal transduction, and the immune response.

##### Protein kinases, catalyc subunits:

Protein kinases, catalytic subunit play a role in various cellular processes, including division, proliferation, apoptosis, and differentiation. They are mainly proteins that modify other ones by chemically adding phosphate groups to them. This usually results in a functional change of the target protein by changing enzyme activity, cellular location, or association with other proteins. The catalytic subunits of protein kinases are highly conserved, and several structures have been solved, leading to large screens to develop kinase-specific inhibitors for the treatments of a number of diseases.

#### The protein data bank

In order to assess the scalability of PROTNN to large scale real-world applications, we evaluate the runtime of our approach on the entire Protein Data Bank (PDB) [4] which contains the list of all known protein 3D-structures. We use 94126 structures representing all the available protein 3D-structures in the PDB by the end of July 2014.

### Protocol and settings

Experiments were conducted on a *CentOS* Linux workstation with an Intel core-i7 CPU at 3.40 GHz, and 16.00 GB of RAM. All the experiments are performed in a single process mode with no parallelization. To transform protein into graph, we used a *δ* value of 7Å. The evaluation measure is the classification accuracy, and the evaluation technique is Leave-One-Out (LOO) where each dataset is used to create *N* classification scenarios, where *N* is the number of proteins in the dataset. In each scenario, a reference protein is used as a query instance and the rest of the dataset is used as reference. The aim is to correctly predict the class of the query protein. The classification accuracy for each dataset is averaged over results of all the *N* evaluations.

## Results and discussion

### PROTNN classification results

#### Results using different distance measures

**Fig. 2:**
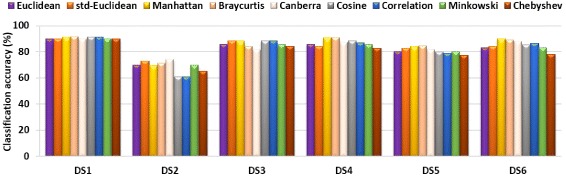
Classification accuracy of PROTNN using different distance measures

The classification algorithm of PROTNN supports any user-defined distance measure. In this section, we study the effect of varying the distance measure on the classification accuracy of PROTNN. We fixed *k*=1, and we used nine different well-known distance measures namely *Euclidean*, *standardized Euclidean* (std-euclidean), *Cosine*, *Manhattan*, *Correlation*, *Minkovski*, *Chebyshev*, *Canberra*, and *Braycurtis*. See [32] for a formal definition of these measures. Figure 2 shows the obtained classification results of a LOO evaluation on each of the benchmark datasets using each of the distance measures.

Overall, varying the distance measure did not significantly affect the classification accuracy of PROTNN on the six datasets. Indeed, the standard deviation of the classification accuracy of PROTNN with each distance measure did not exceed 4 % on the six datasets. A ranking based on the average classification accuracy over the six datasets suggests the following descending order: (1) Manhattan, (2) Braycurtis, (3) std-Euclidean, (4) Canberra, (5) Cosine, (6) Euclidean - Minkowski, (8) Correlation, (9) Chebyshev.

#### Results using different numbers of nearest neighbors

**Fig. 3:**
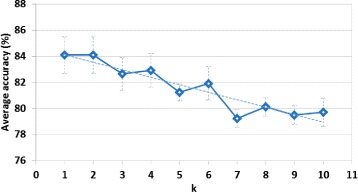
Tendancy of the average accuracy of PROTNN over the six datasets for *k*∈ [1,10]. The *dashed line* represents the linear tendancy of the results

In the following, we evaluate the classification accuracy of PROTNN on each of the six benchmark datasets using different numbers of nearest neighbors *k*∈ [1,10]. For the sake of generalization, we perform the same experiment using each of the top-five distance measures. For simplicity, we only plot the average value of classification accuracy for each value of *k*∈ [1,10] over the six datasets using each of the top-five measures. Note that the standard deviation of ‘ value of *k* did not exceed 2 %. Figure 3 shows the obtained results. The number of nearest neighbors *k* has a clear effect on the accuracy of PROTNN. The results suggest that the “optimal” value of *k*∈ {1,2}. The overall tendency shows that the accuracy decreases with higher values of *k*. This is due to the structural similarity that a query protein may share with other evolutionary close proteins that could belong to the same structural class but exert different functions. High values of *k* engender considering too many neighbors which may causes a misclassification. However, it is worth noting that for datasets with low intra-class similarity among protein structures, PROTNN could need a higher value of *k*.

#### Analysis of the used attributes

In the following, we study the importance of the used attributes in order to identify the most informative ones. We follow the Recursive Feature Elimination (RFE) [33] using PROTNN as the classifier. In RFE, one feature is removed at each iteration, where the remaining features are the ones that best enhance the classification accuracy. We stop the pruning when no further enhancement is observed or no more features are left. The remaining features constitute the optimal subset for that context. In Table 2, we record the ranking of the used attributes in our experiments. For more generalization, RFE was performed on each of the six datasets using a combination of each of the top-five distance measures and each of the top-five values of *k*. The total number of RFE experiments is 150. For each attribute, we count the total number of times it appeared in the optimal subset of attributes. A score of $\frac {total\textit {}count}{number\textit {}of\textit {}experiments}$ is assigned to each attribute according to its total count.

**Table 2:** Empirical ranking of the structural and topological attributes

Data	Attributes																	
	A1	A2	A3	A4	A5	A6	A7	A8	A9	A10	A11	A12	A13	A14	A15	A16	A17	A18
DS1	0	2	1	6	2	1	5	9	10	1	2	8	6	13	16	17	12	17
DS2	8	12	15	16	18	4	9	16	23	17	9	21	11	14	25	17	23	9
DS3	8	13	2	6	17	10	16	11	11	4	8	18	21	2	21	23	9	18
DS4	4	7	21	17	20	6	11	17	16	7	2	14	21	22	20	21	24	17
DS5	12	12	8	10	12	5	7	7	17	17	7	23	23	9	20	9	19	18
DS6	5	11	9	8	11	6	14	14	13	6	1	17	14	18	24	10	17	13
Total	37	57	56	63	80	32	62	74	90	52	29	**101**	**96**	78	126	**97**	**104**	92
Score	0.25	0.38	0.37	0.42	0.53	0.21	0.41	0.49	0.6	0.35	0.19	**0.67**	**0.64**	0.52	**0.84**	**0.65**	**0.69**	0.61
Rank	16	13	14	11	8	17	12	10	7	15	18	**3**	**5**	9	**1**	**4**	**2**	6

The boldface numbers highlight the best performance

It is clear that the best subset of attributes is dataset dependent. The five most informative attributes are respectively: A15 (energy), A17 (link impurity), A12 (number of distinct eigenvalues), A16 (neighborhood impurity), and A13 (spectral radius). All spectral attributes showed to be very informative. Indeed, three of them (A15, A12, and A13) ranked in the top-five, and A14 (second largest eigenvalue) ranked in the top-ten (9^*th*^) with a score of 0.52 meaning that for more than half of all the experiments, all spectral attributes were selected in the optimal subset of attributes. Unsurprisingly, A11 (percentage of end points) ranked last with a very low score. This is because proteins are dense molecules and thus very few nodes of their respective graphs will be end points (extremity amino acids in the primary structure with no spatial links). Label attributes also showed to be very informative. Indeed, A17, A16, and A18 (label entropy) ranked respectively 2 ^*nd*^, 4^*th*^, and 6^*th*^ with scores of more than 0.61. This is due to the importance of the distribution of the types of amino acids and their interactions. Both have to follow a certain harmony in order to produce a particular structural form (for instance an *α*-helix or a *β*-sheet) and to exert a specific function. A9 (closeness centrality), A5 (average clustering coefficient) and A8 (effective radius) ranked in the top-ten with scores of more than 0.5 (A8 scored 0.49 ≃ 0.5). However, all A1 (number of nodes), A2 (number of edges), A3 (average degree), A4 (density), A6 (average effective eccentricity), A7 (effective diameter), and A10 (percentage of central nodes) scored less than 0.5. This is because each one of them is represented by one of the top-ten attributes and thus presents a redundant information. A6 and A9 are both expressed based on all shortest paths of the graph. Both A7 and A8 are expressed based on A6. A10 is expressed based on A8 and thus on A6 too. A1, A2, A3, and A4 are all highly correlated to A5.

#### Analysis of the used classifier

In this section, we perform a comparative analysis on the usage of the principle of KNN classifier in PROTNN versus using another classifier. We chose the Support Vector Machine (SVM) [34] for comparison and we term this approach PROTSVM. We use PROTSVM with a linear kernel SVM (PROTSVM(linear)) than with a non-linear RBF (*Radial Basis Function*) kernel (PROTSVM(rbf)). Table 3 shows the accuracy results of PROTSVM(linear), PROTSVM(rbf) and PROTNN (using the std-Euclidean distance and *k*=1). All the three approaches are used with RFE. We notice that PROTNN scored better than both PROTSVM(linear) and PROTSVM(rbf) on the six datasets with an average classification accuracy of 0.93 compared to 0.81 and 0.74 respectively for PROTSVM(linear) and PROTSVM(rbf).

**Table 3:** Accuracy comparison of PROTNN and PROTSVM

Dataset	Classification approach		
	ProtNN	ProtSVM(linear)	ProtSVM(rbf)
DS1	**0.97**	0.88	0.83
DS2	**0.8**	0.68	0.56
DS3	**0.96**	0.87	0.78
DS4	**0.97**	0.80	0.82
DS5	**0.9**	0.79	0.73
DS6	**0.96**	0.84	0.72
Avg. accuracy^1^	**0.93 ±0.06**	0.81 ±0.07	0.74 ±0.1

^1^Average classification accuracy of each classification approach over the six datasets. The boldface numbers highlight the best performance

#### Comparison with other classification techniques

We compare our approach with multiple state-of-the-art approaches for protein structure classification namely: sequence alignment-based classification (using Blast [8]), structural alignment-based classification (using Combinatorial Extension (CE) [12], Sheba [13], and FatCat [14]), and substructure(subgraph)-based classification (using GAIA [30], LPGBCMP [31], and D&D [17]). For sequence and structural alignment-based classification, we align each protein against all the rest of the dataset. We assign to the query protein the class of the reference protein with the best hit score. For the substructure-based approaches, all the selected approaches are mainly for mining discriminative subgraphs. LPGBCMP is used with *max*_*var*_=1 and *d*=0.25 for, respectively, feature consistency map building and overlapping. In [31], LPGBCMP outperformed several other approaches from the literature including LEAP [35], gPLS [36], and COM [29] on the classification of the same six benchmark datasets. GAIA showed in [30] that it outperformed other state-of-the-art approaches namely COM and graphSig [37]. D&D have showed in [17] that it also outperformed COM and graphSig, and that it is highly competitive to GAIA. For all these approaches, the discovered substructures are considered as features for describing each example of the original data. The constructed description matrix is used for training in the classification. For our approach, we show the classification accuracy results of PROTNN with RFE using std-Euclidean distance. We also show the best results of PROTNN (denoted PROTNN*) with RFE using each of the top-five distance measures. We use *k*=1 for both PROTNN and PROTNN*. Table 4 shows the obtained results.

**Table 4:** Accuracy comparison of PROTNN with other classification techniques

Dataset	Classification approach								
	Blast	Sheba	FatCat	CE	LPGBCMP	D&D	GAIA	ProtNN	ProtNN*
DS1	0.88	0.81	**1**	0.45	0.88	0.93	**1**	0.97	0.97
DS2	0.82	0.86	**0.89**	0.49	0.73	0.76	0.66	0.8	**0.89**
DS3	0.9	0.95	0.84	0.59	0.90	0.96	0.89	0.96	**0.97**
DS4	0.76	0.92	**1**	0.46	0.9	0.93	0.89	0.97	0.97
DS5	0.86	**0.99**	0.94	0.76	0.87	0.89	0.72	0.9	0.94
DS6	0.78	**1**	0.94	0.81	0.91	0.95	0.87	0.96	0.96
Avg. accuracy^1^	0.83 ±0.05	0.92 ±0.07	0.94 ±0.06	0.59 ±0.15	0.86 ±0.06	0.9 ±0.07	0.84 ±0.12	0.93 ±0.06	**0.95 ±0.03**
Avg. distances^2^	0.14 ±0.07	0.05 ±0.07	0.04 ±0.05	0.38 ±0.15	0.11 ±0.03	0.7 ±0.04	0.14 ±0.09	0.05 ±0.03	**0.02 ±0.01**
Rank	8	4	2	9	6	5	7	3	**1**

^1^Average classification accuracy of each classification approach over the six datasets

^2^Average of the distances between the accuracy of each approach and the best obtained accuracy with each dataset

The boldface numbers highlight the best performance

The alignment-based approaches FatCat and Sheba outperformed CE, Blast, and all the subgraph-based approaches. Indeed, FatCat scored best with three of the first four datasets and Sheba scored best with the two last datasets. Except CE, all the other approaches scored on average better than Blast. This shows that the spatial information constitutes an important asset for protein classification by emphasizing structural properties that the primary sequence alone do not provide. For the subgraph-based approaches, D&D scored better than LPGBCMP and GAIA on all cases except with DS1 where GAIA scored best. On average, PROTNN* ranked first with the smallest distance between its results and the best obtained accuracies with each dataset. This is because PROTNN considers both structural information, and hidden topological properties that are omitted by the other approaches. However, all the top four classification methods, namely PROTNN*, FatCat, PROTNN (without parameter optimization) and Sheba, have shown close and very competitive classification results.

In order to make the classification evaluation more challenging we construct a seventh dataset out of the previous six benchmark datasets. This dataset contains seven classes that represent the six positive classes as well as a seventh class that contains all the negative instances from the six benchmark datasets. The fusion of all the negatives into a single large class makes the dataset imbalanced with 29, 33, 38, 38, 35 and 41 instances respectively for the six first classes and 214 instances for the seventh class. This makes the classification even more challenging. We evaluate the classification performance of our approach (namely PROTNN and PROTNN*) compared to FatCat and CE which are the structural alignment approaches used in the PDB website^1^. The classification results on this dataset were 0.53, 0.84, 0.88 and 0.95 respectively for CE, PROTNN, PROTNN* and FatCat. Although FatCat showed a better performance than our approach on this dataset, overall all the approaches did not show a large variation compared to the results on the six first datasets. By counting these results with those on Table 4, both PROTNN* and FatCat have equivalent average classification accuracy of respectively 0.94 ±0.03 and 0.94 ±0.07 on all the datasets, while CE and PROTNN respectively scored 0.58 ±0.15 and 0.91 ±0.07.

### Scalability and runtime analysis

**Fig. 4:**
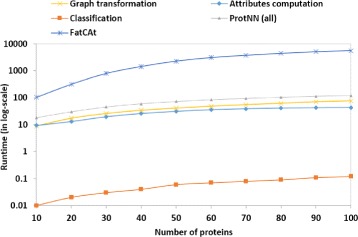
Runtime comparison in log-scale of PROTNN and FatCat. The runtime of PROTNN is separated for the main steps. ProtNN(all) is the sum of its three steps: Graph transformation, Attributes computation and Classification

Besides being accurate, an efficient protein 3D-structure classification approach has to be very fast in order to provide practical usage that meets the increasing load of data in real-world applications. In this section, we study the runtime of our approach and FatCat, the most competitive approach according to our previous comparative experiments. We analyze the variation of runtime for both approaches with increasing number of proteins ranging from 10 to 100 3D-structures with a step-size of 10. In Fig. 4, we report the runtime results in log_10_-scale. A huge gap is clearly observed between the runtime of PROTNN and that of FatCat. The gap gets larger with higher numbers of proteins. Indeed, FatCat took over 5570 s with the 100 proteins while PROTNN (all) did not exceed 118 s for the same set which means that our approach is 47x faster than FatCat on that experiment. The average runtime of graph transformation of PROTNN was 0.8 s and that of the computation of attributes was 0.6 s for each protein. The total runtime of similarity search and classification for PROTNN was only 0.1 on the set of 100 proteins. Note that in real-world applications, the preprocessing (graph transformation and attribute computation) of the reference database is performed only once and the latter can be updated with no need to recompute the existing values. This ensures computational efficiency and easy extension of our approach.

#### Scalability to a PDB-wide classification

We further evaluate the scalability of PROTNN in the classification of the entire Protein Data Bank (described in The protein data bank). We also show the runtime for FatCat and CE (the structural alignment approaches used in the PDB website). We recall that the experiments are on a single process mode with no parallelization for all the approaches. Note that in the PDB website, the structural alignment is whether pre-computed for structures of the database, or only performed on a sub-sample of the PDB for customized or local files. Table 5 shows the obtained results. It is clear that the computation of attributes is the most expensive part of our approach as some attributes are very complex. However, building the graph models and the computation of attributes represent the preprocessing step and are only performed once for the reference database. The classification step took almost three hours with an average runtime of 0.1 s for the classification of each protein against the entire PDB. All PROTNN runtime was less than a week with an average runtime of 5.9 s for the preprocessing and classification of each protein 3D-structure against the entire PDB. On the other hand, both FatCat and CE did not finish running within two weeks. We computed the average runtime for each approach on the classification of a random sample of 100 proteins against all the PDB. On average FatCat and CE took respectively more than 42 and 32 h per protein making our approach faster than both approaches with thousands orders of magnitude on the classification of a 3D-structure against the entire PDB.

**Table 5:** Runtime results of PROTNN, FatCat and CE on the entire Protein Data Bank

Task	Total runtime^1^	Runtime^1^/protein
Building graph models	23h:9m:57s	0.9s
Computation of attributes	5d:8h:12m:29s	4.9s
Classification	2h:55m:15s	0.1s
ProtNN (all)	6d:10h:17m:41s	5.9s
FatCat	Forever^2^	1d:18h:31m:35s^3^
CE	Forever^2^	1d:8h:37m:34s^3^

^1^The runtime is expressed in terms of days:hours:minutes:seconds

^2^The program did not finish running within two weeks

^3^The average runtime of randomly selected 100 proteins

## Conclusion

In this paper, we proposed PROTNN, a new fast and accurate approach for protein 3D-structure classification. We defined a graph transformation and embedding model that incorporates explicit as well as hidden structural and topological properties of the 3D-structure of proteins. We successfully implemented the proposed model and we experimentally demonstrated that it allows to classify protein 3D-structures efficiently. Empirical results of our experiments showed that considering structural information constitutes a major asset for an accurate classification of proteins. They also showed that the alignment-based classification as well as subgraph-based classification present very competitive approaches. Yet, as the number of pairwise comparisons between proteins grows tremendously with the size of dataset, enormous computational costs would be the results of more detailed models. Here, we highlight that PROTNN could accurately classify multiple benchmark datasets from the literature with very low computational costs. With all large-scale studies, it is an asset that PROTNN scales up to a PDB-wide dataset in a single-process mode with no parallelization, where it outperformed state-of-the-art approaches with thousands order of magnitude in runtime on classifying a 3D-structure against the entire PDB. In future works, we aim to study and integrate more attributes in our model in order to further enhance the accuracy of our classification system.

## Endnote

^1^http://www.rcsb.org/pdb/.

## References

[1] Brenner SE, Levitt M (2000). Expectations from structural genomics. Protein Sci.

[2] Lee D, Redfern O, Orengo C (2007). Predicting protein function from sequence and structure. Nat Rev Mol Cell Biol.

[3] Molloy K, Van MJ, Barbara D, Shehu A (2014). Exploring representations of protein structure for automated remote homology detection and mapping of protein structure space. BMC Bioinformatics.

[4] Berman HM, Westbrook JD, Feng Z, Gilliland G, Bhat TN, Weissig H, Shindyalov IN, Bourne PE (2000). The protein data bank. Nucleic Acids Res.

[5] Andreeva A, Howorth D, Chandonia JM, Brenner SE, Hubbard TJP, Chothia C, Murzin AG (2008). Data growth and its impact on the scop database: new developments. Nucleic Acids Res.

[6] Sillitoe I, Lewis TE, Cuff AL, Das S, Ashford P, Dawson NL, Furnham N, Laskowski RA, Lee D, Lees JG, Lehtinen S, Studer RA, Thornton JM, Orengo CA (2015). CATH: comprehensive structural and functional annotations for genome sequences. Nucleic Acids Res.

[7] Meysman P, Zhou C, Cule B, Goethals B, Laukens K (2015). Mining the entire protein databank for frequent spatially cohesive amino acid patterns. BioData Mining.

[8] Altschul S, Gish W, Miller W, Myers E, Lipman D (1990). Basic local alignment search tool. J Mol Biol.

[9] Jensen LJ, Gupta R, Stærfeldt HH, Brunak S (2003). Prediction of human protein function according to gene ontology categories. Bioinformatics.

[10] Cai CZ, Han LY, Ji ZL, Chen X, Chen YZ (2003). Svm-prot: web-based support vector machine software for functional classification of a protein from its primary sequence. Nucleic Acids Res.

[11] Jaramillo-Garzón JA, Gallardo-Chacón JJ, Castellanos-Domínguez CG, Perera-Lluna A (2013). Predictability of gene ontology slim-terms from primary structure information in embryophyta plant proteins. BMC Bioinformatics.

[12] Shindyalov IN, Bourne PE (1998). Protein structure alignment by incremental combinatorial extension of the optimum path. Protein Eng.

[13] Jung J, Lee B (2000). Protein structure alignment using environmental profiles. Protein Eng.

[14] Ye Y, Godzik A (2003). Flexible structure alignment by chaining aligned fragment pairs allowing twists. Bioinformatics.

[15] Budowski-Tal I, Nov Y, Kolodny R (2010). Fragbag, an accurate representation of protein structure, retrieves structural neighbors from the entire pdb quickly and accurately. Proc Nat Acad Sci.

[16] Borgwardt KM, Ong CS, Schönauer S, Vishwanathan SVN, Smola AJ, Kriegel H. Protein function prediction via graph kernels. In: Proceedings Thirteenth International Conference on Intelligent Systems for Molecular Biology 2005, Detroit, MI, USA, 25–29 June 2005. p. 47–56.

[17] Zhu Y, Yu JX, Cheng H, Qin L (2012). Graph classification: a diversified discriminative feature selection approach. 21st ACM International Conference on Information and Knowledge Management.

[18] Dhifli W, Saidi R, Mephu Nguifo E (2014). Smoothing 3D protein structure motifs through graph mining and amino-acids similarities. J Comput Biol.

[19] Wolpert D, Macready WG (1997). No free lunch theorems for optimization. IEEE Trans Evol Comput.

[20] Huan J, Bandyopadhyay D, Wang W, Snoeyink J, Prins J, Tropsha A (2005). Comparing graph representations of protein structure for mining family-specific residue-based packing motifs. J Comput Biol.

[21] Gibert J, Valveny E, Bunke H (2010). Graph of words embedding for molecular structure-activity relationship analysis. Progress in Pattern Recognition, Image Analysis, Computer Vision, and Applications. Lecture Notes in Computer Science.

[22] Watts DJ, Strogatz SH (1998). Collective dynamics of ‘small-world’ networks. Nature.

[23] Reka Albert HJ, Barabasi AL (1999). Internet: Diameter of the world-wide web. Nature.

[24] Luo B, Wilson RC, Hancock ER (2003). Spectral embedding of graphs. Pattern Recognit.

[25] Leskovec J, Kleinberg J, Faloutsos C (2005). Graphs over time: densification laws, shrinking diameters and possible explanations. Eleventh ACM SIGKDD International Conference on Knowledge Discovery in Data Mining.

[26] Li G, Semerci M, Yener B, Zaki MJ (2011). Graph classification via topological and label attributes. 9th Workshop on Mining and Learning with Graphs (with SIGKDD). MLG’11.

[27] Li G, Semerci M, Yener B, Zaki MJ (2012). Effective graph classification based on topological and label attributes. Stat Anal Data Mining.

[28] Mitchell TM (1997). Machine Learning.

[29] Jin N, Young C, Wang W. Graph classification based on pattern co-occurrence. In: ACM International Conference on Information and Knowledge Management: 2009. p. 573–82.

[30] Jin N, Young C, Wang W (2010). GAIA: graph classification using evolutionary computation. Proceedings of the 2010 ACM SIGMOD International Conference on Management of Data.

[31] Fei H, Huan J. Boosting with structure information in the functional space: an application to graph classification. In: ACM Knowledge Discovery and Data Mining Conference (KDD): 2010. p. 643–52.

[32] Sergio J, Rojas G, Blanco-Silva FJ, Christensen EA (2015). Learning SciPy for Numerical and Scientific Computing - Second Edition. Community experience distilled.

[33] Guyon I, Weston J, Barnhill S, Vapnik V (2002). Gene selection for cancer classification using support vector machines. Mach Learn.

[34] Vapnik V, Cortes C (1995). Support-vector networks. Machine Learn.

[35] Yan X, Cheng H, Han J, Yu PS (2008). Mining significant graph patterns by leap search. Proceedings of the ACM SIGMOD International Conference on Management of Data. SIGMOD.

[36] Saigo H, Krämer N, Tsuda K. Partial least squares regression for graph mining. In: ACM Knowledge Discovery and Data Mining Conference (KDD): 2008. p. 578–86.

[37] Ranu S, Singh AK. Graphsig: A scalable approach to mining significant subgraphs in large graph databases. In: IEEE 25th International Conference on Data Engineering: 2009. p. 844–55.

